# Southern Dental Association

**Published:** 1887-10

**Authors:** 


(TO BE CONTINUED.)

---

### SOUTHERN DENTAL ASSOCIATION.

### NINETEENTH ANNUAL SESSION AT OLD POINT COMFORT, VA., 1887.

REPORTED FOR THE INDEPENDENT PRACTITIONER, BY "MRS. M. W. J."

The Southern Dental Association convened in nineteenth annual session at Old Point Comfort, Va., Tuesday, August 30, 1887.

Ample accommodations had been secured in the Hygeia Hotel, and the soldiery of Fortress Monroe, adjacent, furnished abundant material for every class of dental operations for the clinics.

The President, Dr. W. H. H. Thackston, presided at every session.

The first session was called to order at 10 A. M. The address of welcome was delivered by Dr. V. Edward Turner, of North Carolina, and the response by Dr. J. H. Prewitt, of Kentucky.

Dr. Ernst Sjoberg, of Stockholm, Sweden, responded on behalf of the foreign members of the profession present. Visiting dentists from abroad, and those from the north, east and west, were invited to participate in the discussions.

The President then delivered his annual address.

At the session of 3 P. M., after some routine business, on motion of Dr. J. B. Hodgkin, of Baltimore, a committee of three was appointed to draft resolutions expressive of the sense of loss sustained by the Southern Association in the death of Dr. J. R. Walker, of New Orleans, one of its oldest and most active members, and one of the founders of the Association.

After announcements from the Committee of Arrangements, the

Committee on Clinics, the Committee on Appliances, etc., the meeting adjourned to 10 A. M. of Wednesday, August 31st, when Dr. A. E. Baldwin, of Chicago, Ill., as the special order of the day, read a paper entitled

IMMEDIATE ROOT FILLING.

The writer was not an advocate for pulp-capping, when the pulp is truly exposed, and judges of the merits of the system from the remarks of many patients upon whom the operation had been performed, and who were subsequently having abscesses treated or roots filled in teeth whose pulps had been saved (?) by capping. He believed that the interests of the patient were best subserved by devitalization and immediate root filling. He said that the usefulness of the pulp has been mainly accomplished when the tooth is fully developed, and he would strive more earnestly to save a pulp for a child than for an adult. In the majority of cases of abscesses, the roots are best filled immediately, thus removing the cause of the trouble, which lies in the condition of the pulp-chamber or root-canals. If the difficulty is due to necrosed surroundings, it can be better treated from the outside than through the root-canals, and nothing is gained by leaving them open.

After applying the rubber dam and cleaning out the pulp-chamber and root-canals as thoroughly as possible, his practice is to wipe them out with a ninety-five per cent. solution of carbolic acid, and then with alcohol, because of its affinity for the water in the tooth substance.

His main reliance is on the next step, which is that of the thorough drying of the canals, and the desiccation of the contents of the dentinal tubules, by means of hot air, never attempting to fill till he is morally sure that the tooth substance is thoroughly dry. Gutta-percha solution is then pumped in the canals until they are thoroughly filled, using in this process a broach made of piano wire, roughened by rolling it under a fine metal file, and wrapped with a fine shred of cotton. A piece of heated base-plate gutta-percha is placed over the opening to the root-canals, and the contents forced into the uttermost limits of the dry canals, and even into the mouths of the tubules. The walls being perfectly dry, the gutta-percha will cling tenaciously to them, instead of shrinking away, as is the case when there is moisture. The writer of the paper waits for a week or ten days after devitalization before removing the dead tissue, allowing it time to slough from the living tissue, thus saving pain to the patient. He did not claim to remove all the debris from all canals, some being so tortuous that they cannot be gotten at. He also thinks that the importance of micro-organisms has been greatly exaggerated. He does not accept the dictum that there is never any pus-formation save in the presence of micro-organisms, or atmospherical germs, believing the contrary proved by the formation of pus in a felon, or an acute synovitis of the knee-joint. He does not think the entire removal of all the debris from even the smallest canals necessary, provided it be desiccated and hermetically sealed. He does not commend the use of a long list of new remedies, believing that with earnest study of their capabilities and adaptabilities, a few, carefully selected, will be found to meet all our wants. In conclusion, he urged each one to reason and think for himself, and not be a blind follower of any one. In this way we shall grow, expand, and progress onward and upward toward the goal of perfection.

*Dr. M. C. Marshall,* Little Rock, Arkansas—Read a paper entitled

CONSERVATISM IN SELECTING FILLING MATERIALS, and the two papers were discussed together.

Dr. Marshall spoke of the introduction of cohesive gold, and the delight with which the welding principle was hailed. To best serve his patients, the operator must adopt a conservative course. If his judgment conflicts with the ideas of his patient, he must have the courage of his convictions. To be a successful operator, he must have the mechanical skill to introduce a filling properly, an artistic eye to reproduce nature, and scientific knowledge to determine what material is best for the case in hand. He must consider the constitutional tonicity and the age of the patient, and the position of the cavity. Duty must transcend any hobby, and the æsthetic must not dominate the practical.

In the discussion which followed the reading of the papers, Dr. Baldwin was asked why he used the ninety-five per cent. solution of carbolic acid. He replied that pure carbolic acid was always in crystals ; the water of deliquescence formed in warming the crystals is the ninety-five per cent. solution which is used in wiping out canals, etc.

*Dr. Winckler,* of Georgia—Approved the suggestion of drying to desiccation, but preferred lead points or carbolized wood, and gutta-percha points for root-canal fillings. He thought the immediate filling applicable only to roots freshly devitalized, for which purpose he preferred knocking them out with the wooden peg. It can be done very quickly, is almost painless when skillfully done, and the tooth requires no treatment, after removal of the fibrous tissue, beyond washing out with alcohol. Dormant dead pulps require treatment with peroxide of hydrogen till ebullition ceases, when he applies a paste of iodoform and creosote until the next day.

*Dr. J. C. Storey,* Dallas, Texas—Did not pretend to fill all roots. He had not seen any of the drills that could follow tortuous canals and stop right at the apex. He used oxy-chloride of zinc for root fillings.

*Dr. C. E. Kells, Jr.,* of New Orleans—Does not think the contents of root-canals which are too fine to admit a nerve bristle will do any harm. He uses carbolized wood points for the apex, and fills with Guillois' cement.

*Dr. W. H. Morgan,* of Nashville—Drills all root-canals, cutting out the dentine, and with it the .tubuli and contents. He doubts the possibility of the desiccation spoken of. If it could be done it would effectually prevent decomposition, as fluids are essential to germs, and germs are essential to putrefraction.

*Dr. T. T. Moore,* of Columbia, S. C.—Pumps in liquid gutta-percha, and then drives in a lead or wood point, forcing the gutta-percha in all directions.

*Dr. W. H. Richards,* of Knoxville, Tennessee—When forced to devitalize, applies arsenical paste, removing the pulp four hours after making the application, when the pulp is congested and anæsthetized. At that point the removal is effected almost without pain. He then fills with a paste of ninety-five per cent. solution of carbolic acid and iodoform, which he leaves in the canals for twenty-four hours, to prevent septic action in the tubuli.

*Dr. Sjoberg,* of Stockholm, Sweden—Said that his practice, in cases where the roots were liable to be very difficult of access, and the pulp inflamed but not reduced to a formless mass, was to apply arsenical paste, removing only the coronal bulb of the pulp, placing over the opening to the root-canals (the contents of which were left undisturbed) a paste of carbolic acid and oxide of zinc, capping over this with a platium disk, and filling the pulp-chamber with cement. This operation was termed amputation of the pulp, and was usually very successful. It is used only in very bad cases, in which the canals cannot be washed to clean and fill.

*Dr. McKellops,* of St. Louis—Said that with all our best efforts there was still a lamentable want of success. No man would honestly claim to clean and fill all root-canals.

Abscesses would sometimes occur, even in the cases where we had done our most thorough, careful work, and when we think we have successfully filled to the very apex. We do not know the causes of these failures—it may be microbes, it may be chemical action. Our best students are at work on the question. The "successfully" filled roots that we extract, with their contents of gold, and lead, and wood, and gutta-percha, and oxy-chloride, and even cotton, tell the tale better than the words of the operator who "succeeds" every time in filling even the most tortuous canals. He commended highly the gold broaches introduced by Dr. Herbst last year.

*Dr. Beach,* of Clarksville, Tennessee—Did not approve of the idea expressed in the paper, of waiting ten days before removing the devitalized pulp, which would then be an offensive mass, with perhaps a very sensitive stump at the apex. He much preferred the plan of stabbing out the freshly exposed pulp with a peg of wood, as being much less painful to the patient and more promptly done. After an experience of seventeen years with lead points for root fillings, he preferred that to any other material. It was soft and would take the shape of the canal, while even if driven through the apex it will be encysted and cause no irritation.

*Dr. A. Eubanks,* of Birmingham, Alabama—Dries root-canals and cavities with alcohol, followed by chloroform.

*Dr. Stockton,* of New Jersey—Thought the liability to subsequent trouble from capped pulps almost inevitable. They were much safer taken out. If a patient was forwarned that it would "hurt a little," his anticipations would be so much worse than the reality that he would be pleasantly surprised at the ease of the operation of knocking out the freshly exposed pulps. He hoped the dental manufacturers would give us some means of holding compressed heated air in a reservoir, as it was invaluable in many cases.

*Dr. Allport,* of Chicago—Wished to thank Dr. Baldwin for his excellent paper. He would make but one criticism, which was in using the term "dead teeth" when pulpless teeth were intended. The tooth is not an isolated organ, but has a vital connection with the rest of the system, and even though the pulp be dead, the cementum is still nourished by the peridentium, with some anastamosis between the canaliculi of the cementum and the tubuli of the dentine. If the tooth was so treated that the contents of the tubuli would not decompose, it made but little difference what filling material was used. Heat was the best agent for the removal of moisture and gases, applied, not by means of the hot-air syringe, but by suitable instruments, the best being one holding a broach connected with a large bulb, the latter to be heated in the flame of the alcohol lamp, the heated broach to be applied to all parts of the cavity and root-canals till the liquid contents are boiled out. Peroxide of hydrogen will expel anything more that may remain. The tooth which is thus dried and purified, and with the apex sealed, is safe. The term alveolar abscess is misapplied in nineteen cases out of twenty. A true alveolar abscess occurs only where the gases form within the tubuli and pass through the cementum into the peridental membrane. Gas escaping from the apex follows the track of circulation, and does not in one case in a hundred form a true alveolar abscess. Because of the probable shrinkage of gutta percha, he prefers the semi-fluid oxy-chloride of zinc, coaxing it up into the canals with gold wrapped on a broach till it is forced in all directions, when the broach is withdrawn and the gold left in the canal.

*Dr. McKellops*—Said he would be willing to go to Chicago to see his young friend, Allport, make a tooth hot enough to "hiss" in the mouth.

*Dr. Morgan*—Said that the instrument with bulb and broach was identical with one used forty years ago to destroy pulps, burning them out as with fire.

*Dr. Freeman,* of Tennessee—Wished to ask those who had never used lead points in root-filling to go home and give it a fair trial and report their failures, if any, at the next annual meeting.

After some further discussion the subject was passed, and the Association adjourned to meet at 3 P. M.

